# The impact of influenza vaccination on surgical outcomes in COVID-19 positive patients: An analysis of 43,580 patients

**DOI:** 10.1371/journal.pone.0281990

**Published:** 2023-03-10

**Authors:** Susan M. Taghioff, Benjamin R. Slavin, Shefali Mehra, Tripp Holton, Devinder Singh

**Affiliations:** 1 Division of Plastic & Reconstructive Surgery, University of Miami Miller School of Medicine, Miami, Florida, United States of America; 2 Department of Surgery, Luminis Health-Anne Arundel Medical Center, Annapolis, Maryland, United States of America; University of Pittsburgh, UNITED STATES

## Abstract

**Background:**

Multiple recent studies suggest a possible protective effect of the influenza vaccine against severe acute respiratory coronavirus 2 (SARS-CoV-2). This effect has yet to be evaluated in surgical patients. This study utilizes a continuously updated federated electronic medical record (EMR) network (TriNetX, Cambridge, MA) to analyze the influence of the influenza vaccine against post-operative complications in SARS-CoV-2-positive patients.

**Methods:**

The de-identified records of 73,341,020 patients globally were retrospectively screened. Two balanced cohorts totaling 43,580 surgical patients were assessed from January 2020-January 2021. Cohort One received the influenza vaccine six months-two weeks prior to SARS-CoV-2-positive diagnosis, while Cohort Two did not. Post-operative complications within 30, 60, 90, and 120 days of undergoing surgery were analyzed using common procedural terminology(CPT) codes. Outcomes were propensity score matched for characteristics including age, race, gender, diabetes, obesity, and smoking.

**Results:**

SARS-CoV-2-positive patients receiving the influenza vaccine experienced significantly decreased risks of sepsis, deep vein thrombosis, dehiscence, acute myocardial infarction, surgical site infections, and death across multiple time points(p<0.05, Bonferroni Correction p = 0.0011). Number needed to vaccinate (NNV) was calculated for all significant and nominally significant findings.

**Conclusion:**

Our analysis examines the potential protective effect of influenza vaccination in SARS-CoV-2-positive surgical patients. Limitations include this study’s retrospective nature and reliance on accuracy of medical coding. Future prospective studies are warranted to confirm our findings.

## Introduction

With over 279 million cases and 5.3 million deaths globally, the current severe acute respiratory syndrome coronavirus 2 (SARS-CoV-2) pandemic continues to alter daily life [1]. Given the relative paucity of clinical information coupled with the ubiquitous spread of SARS-CoV-2, the medical community has been challenged to provide answers. Instrumental in finding solutions to combat the current pandemic is the need for reliable and accurate clinical data. One potential answer is federated electronic medical record (EMR) networks. Such technology can analyze millions of deidentified records in minutes thereby helping to guide future prospective studies during health crises, such as the current pandemic.

One aspect of clinical practice that has been deeply impacted by the COVID-19 pandemic is the field of surgery. Unsurprisingly, non-elective surgical procedures in SARS-CoV-2-postive patients have yielded poor post-operative outcomes with significantly increased morbidity and mortality when compared with SARS-CoV-2-negative patients [2, 3]. Specifically, *The COVID-Surg Collaborative* has published an international cohort study of 1128 SARS-CoV-2 positive patients who underwent surgery, of whom 82% of deaths were attributed to post-operative pulmonary complications; significantly higher than surgical patients negative for SARS-CoV-2 [4].

Given the early indications that SARS-CoV-2-positive status is associated with increased risk for adverse post-operative outcomes, additional insight into ways to combat this effect is essential [2, 3]. With the development of COVID vaccines, there is hope that postoperative outcomes may improve to pre-pandemic levels. However, even with the unprecedented surge in vaccine production, global demand has placed an inevitable strain on the limited supply and distribution around the world. Therefore, a large portion of the global population remains unvaccinated and vulnerable [5].

Recently, several studies have suggested that influenza vaccination is protective against adverse outcomes associated with SARS-CoV-2 including hospitalization, ICU admission, sepsis, stroke, and ED visits [6–8]. Multiple hypotheses regarding the underlying mechanism of influenza vaccination’s potential protective effect against SARS-CoV-2 have been proposed, suggesting an increase in innate immune system acitvation [9–15]. Despite increasing evidence supporting influenza vaccine protection against SARS-CoV-2, this finding has yet to be examined in surgical patients. By retrospectively reviewing over 43,000 de-identified EMRs, this study aims to investigate and characterize the potential protective effect of up-to-date influenza vaccination against various adverse post-operative outcomes in SARS-CoV-2-positive surgical patients.

## Methods

**Fig 1:**
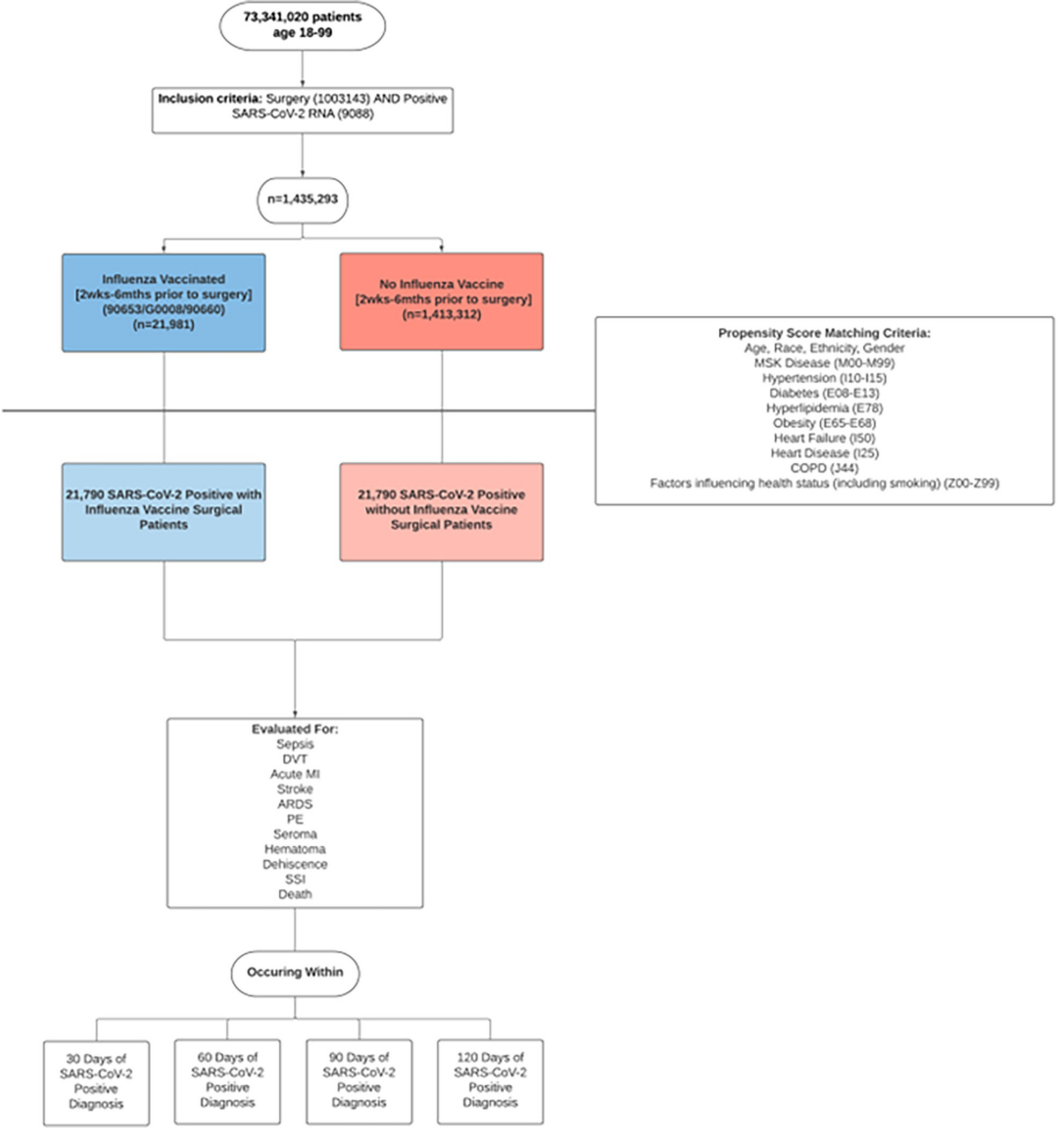
Study design, illustrating methodology and inclusion criteria. Two equally balanced cohorts of 21,790 created using propensity score matching. Various adverse outcomes were analyzed and compared between the influenza vaccinated and non-influenza vaccinated cohorts.

The EMRs of 73,341,020 patients aged 18–99 were retrospectively screened from January 2020-January 2021 in the TriNetX database (TriNetX Inc, Cambridge, MA) (Fig 1). TriNetX is a federated EMR network that aggregates the de-identified medical records of over 73 million patients from 56 participating healthcare organizations into a central, self-updating platform [16].

### Ethics statement

The methodology of this article was reviewed in full by the Institutional Review Board of the University of Miami. Notably, the authors were completely blinded to any identifiable information associated with the EMRs included on the federated network utilized for this analysis. Given the de-identified nature of the individual EMRs and strict Health Information Portability and Accountability Act (HIPAA)-compliant measures put in place by the federated EMR network platform, this study was granted IRB exemption status and therefore the requirement for written consent forms was waived [16].

### Inclusion criteria

Standardized Logical Observations Identifiers Names and Codes (LOINC) codes were used to identify patients who were positive for SARS-CoV-2 (LOINC: 94500–6) while undergoing surgery (LOINC: 1003143). Additionally, Current Procedural Terminology (CPT) codes were used to capture patients who had received influenza vaccination. Specifically, up-to-date influenza vaccination was defined as administration of either the trivalent live intranasal (90660) or inactivated intramuscular influenza vaccine (90653) within two weeks to six months prior to the date of surgery with a SARS-CoV-2 positive diagnosis. This timeframe was established based upon current CDC guidelines which state that full immunity is achieved two weeks from the date of influenza vaccination, with adequate levels of antibody protection lasting approximately six months prior to observation of a waning effect [17]. Any patients who were either outside of the age range or did not undergo surgery from January 2020-January 2021 while SARS-CoV-2-positive were excluded.

### Cohort balancing

Following application of inclusion and exclusion criteria, one cohort of influenza-vaccinated and one cohort of non-influenza vaccinated, SARS-CoV-2-positive surgical patients were created. Propensity score matching was conducted to minimize confounding and increase external validity. Numerous factors were matched between the two cohorts including: age, race, ethnicity, gender, musculoskeletal disease (M00-M99), hypertension (I10-I16), diabetes (E08-E13), hyperlipidemia (E78), obesity (E65-E68), heart failure (I50), heart disease (I25), chronic obstructive pulmonary disease (J44), and factors influencing health status, including smoking, body mass index (BMI), and socioeconomic status (Z00-Z99).

Using the TriNetX online database platform for real-time analyses, we performed propensity score matching to create cohorts consistent with the aforementioned criteria. Propensity score 1:1 balancing was completed via logistic regression utilizing version 3.7 of Python Software Foundation’s *Scikit-Learn* package (Python Software Foundation, Delaware, USA). A greedy nearest neighbor matching algorithm approach was used, setting standard differences to a value of less than 0.1 to indicate appropriate matching. To eliminate record order bias, randomization of the record order in a covariate matrix occurred before matching. Baseline characteristics with a standardized mean difference between cohorts lower than 0.1 was considered well-balanced.

### Outcomes assessed

Propensity score matching resulted in two equally-sized cohorts. Cohort One received influenza vaccination within two weeks to six months prior to undergoing surgery with a SARS-CoV-2 positive diagnosis whereas Cohort Two did not. Post-operative complications were then compared between the two cohorts 30, 60, 90, and 120 days after the index event. The following adverse outcomes were assessed using International Classification of Diseases-10 (ICD-10) codes and included Sepsis (A41.9, T81.44), deep vein thrombosis (DVT) (I82.22, I82.40-I82.89, I82.19), acute myocardial infarction (Acute MI) (I21), stroke (I63), acute respiratory distress syndrome (ARDS) (J80), pulmonary embolism (PE) (I26), surgical site infection (SSI) (T81.41, T81.42, T81.49), dehiscence (T81.30, T81.31), hematoma (L76.32), seroma (L76.34), and death.

### Effect size analysis

**Fig 2:**
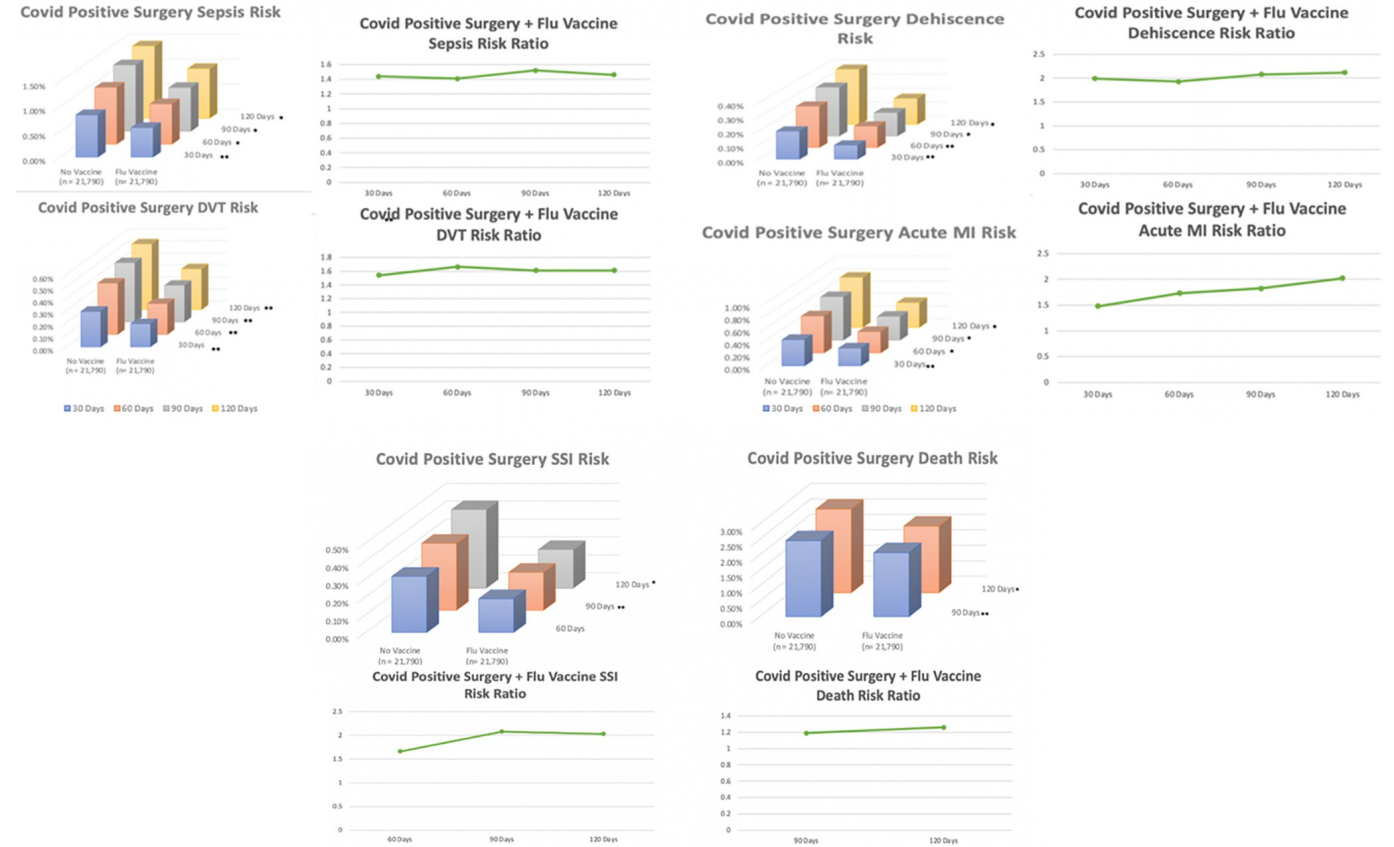
Significantly different (*) and nominally significant (**) risks & risk ratios of post-operative adverse outcomes between influenza vaccinated and non-influenza vaccinated patients within 30–120 days (Fig 2a) and 90–120 days (Fig 2b) of surgery. * denotes statistical significance following Post-Hoc testing for Multiple Hypotheses using Bonferroni Correction to alpha level of p = 0.0011, ** denotes nominally significant values (p < 0.05) that failed to meet statistical significance following Bonferroni correction of p = 0.0011.

Using the TriNetX platform’s *Analytics* function, statistical analysis and logistical regression were performed by comparing indices and relative risks of outcomes only after the successful matching of cohorts with a p-value greater than 0.05. Outcomes for all measures were calculated using 95% confidence intervals (CIs). All p-values were two-sided and the alpha level was set at 0.05. To account for multiple hypothesis testing, post-hoc analysis using a Bonferroni correction of p = 0.0011 was calculated for the 11 adverse outcomes across 4 different time points. Outcomes with p-values less than 0.05 but greater than the Bonferroni correction of p = 0.0011 were deemed to be nominally significant and are indicated as such versus outcomes that were found to be truly significant even following Bonferroni correction (Tables 1–3, Fig 2). Risk ratio (RR) was defined in this study as the ratio of the probability of an adverse post-operative event occurring without history of up-to-date influenza vaccination versus the probability of the adverse post-operative event occurring with history of up-to-date influenza vaccination.

**Table 1:** Distribution of types of surgical procedures undergone by SARS-CoV-2-positive patients included in this analysis who had either received or not received pre-operative influenza vaccination. The distribution between the vaccinated and non-vaccinated cohorts was noted to be equal following propensity score matching and cohort balancing.

**Surgical Procedures on the Digestive System 18.2%**
Surgical procedures on the Colon and Rectum
Surgical Procedures on the Esophagus
Surgical Procedures on the Intestines (Except Rectum)
Surgical Procedures on the Biliary Tract
Surgical Procedures on the Anus
Surgical Procedures on the Pharynx, Adenoids, and Tonsils
Surgical Procedures on the Stomach
Surgical Procedures on the Liver
Surgical Procedures on the Appendix
Surgical Procedures on the Dentoalveolar Structures
Surgical Procedures on the Salivary Gland and Ducts
Surgical Procedures on the Vestibule of the Mouth
Surgical Procedures on the Tongue and Floor of the Mouth
Surgical Procedures on the Palate and Uvula
Surgical Procedures on the Pancreas
**Surgical Procedures on the Musculoskeletal System 16.9%**
General Surgical Procedures on the Musculoskeletal System
Surgical Procedures on the Femur (Thigh Region) and Knee Joint
Surgical Procedures on the Pelvis and Hip Joint
Surgical Procedures on the Foot and Toes
Surgical Procedures on the Hands and Fingers
Surgical Procedures on the Shoulder
Surgical Procedures on the Forearm and Wrist
Surgical Procedures on the Spine (Vertebral Column)
Surgical Procedures on the Leg (Tibia and Fibula) and Ankle Joint
Surgical Procedures on the Head
Surgical Procedures on the Neck (Soft Tissues) and Thorax
Surgical Procedures on the Back and Flank
Surgical Procedures on the Abdomen
**Surgical Procedures on the Cardiovascular System 14.7%**
Surgical Procedures on the Arteries and Veins
Surgical Procedures on the Heart and Pericardium
**Surgical Procedures on the Integumentary System 13.9%**
Destruction Procedures on the Integumentary System
Surgical Procedures on the Nails
Surgical Procedures on the Breast
Surgical Procedures on the Pilonidal Cyst
Other Procedures on the Integumentary System
**Surgical Procedures on the Nervous System 8.2%**
Surgical Procedures on the Spine and Spinal Cord
Surgical Procedures on the Skull, Meninges, and Brain
**Surgical Procedures on the Urinary System 6.9%**
Surgical Procedures on the Bladder
Surgical Procedures on the Kidney
Surgical Procedures on the Urethra
Surgical Procedures on the Ureter
**Surgical Procedures on the Respiratory System 6.5%**
Surgical Procedures on the Larynx
Surgical Procedures on the Trachea and Bronchi
Surgical Procedures on the Accessory Sinuses
Surgical Procedures on the Lungs and Pleura
Surgical Procedures on the Nose
**Surgical Procedures on the Auditory System 4.8%**
Surgical Procedures on the External Ear
Surgical Procedures on the Middle Ear
Surgical Procedures on the Inner Ear
**Surgical Procedures on the Eye and Ocular Adnexa 2.6%**
Surgical Procedures on the Anterior Segment of the Eye
Surgical Procedures on the Posterior Segment of the Eye
Surgical Procedures on the Ocular Adnexa
Surgical Procedures on the Conjunctiva
Surgical Procedures on the Eyeball
**Surgical Procedures on the on the Female Genital System 2.6%**
Surgical Procedures on the Corpus Uteri
Surgical Procedures on the Vagina
Surgical Procedures on the Oviduct/Ovary
Surgical Procedures on the Cervix Uteri
Surgical Procedures on the Vulva, Perineum and Introitus
Surgical Procedures for In Vitro Fertilization
**Surgical Procedures on the Hemic and Lymphatic Systems 2.2%**
Surgical Procedures on the Spleen
**Surgical Procedures on the Male Genital System 1.7%**
Surgical Procedures on the Prostate
Surgical Procedures on the Penis
Surgical Procedures on the Vas Deferens
Surgical Procedures on the Testis
Surgical Procedures on the Epididymis
Surgical Procedures on the Tunica Vaginalis
Surgical Procedures on the Scrotum
Surgical Procedures on the Spermatic Cord
**Surgical Procedures for Maternity Care and Delivery 0.4%**
Vaginal Delivery, Antepartum and Postpartum Care Procedures
Cesarean Delivery Procedures
Introduction Procedures for Maternity Care and Delivery
Repair Procedures for Maternity Care and Delivery
Delivery Procedures After Previous Cesarean Delivery
Abortion Procedures
Other Procedures for Maternity Care and Delivery
**Surgical Procedures on the Endocrine System 0.4%**
Surgical Procedures on the Thyroid Gland

**Table 2:** Results of propensity score matching to balance cohorts.

			Before Matching				After Matching		
Codes	Demographics	Mean ± SD	Patients	% of Cohorts	p-Value	Mean ± SD	Patients	% of Cohorts	p-Value
AI	Age at Index	**46.6 ± 22.1 58.9 ± 21.1**	1,431,993 21,524	100% 100%	<0.0001	59 ± 21 58.9 ± 21.1	21,524 21,524	100% 100%	0.8557
2186-5	Not Hispanic or Latino		1,036,201 18,239	72.361% 84.738%	<0.0001		18,271 18,239	84.887% 84.738%	0.6674
2106-3	White		1,030,138 16,136	71.937% 74.967%	<0.0001		16,221 16,136	75.362% 74.967%	0.3430
F	Female		830, 840 12,795	58.02% 59.445%	<0.0001		12,804 12,795	59.487% 59.445%	0.9296
M	Male		600,719 8,727	41.95% 40.545%	<0.0001		8,715 8,727	40.49% 40.545%	0.9062
2054-5	Black or African American		219,822 3,037	15.351% 14.11%	<0.0001		3,000 3,037	13.938% 14.11%	0.6076
UN	Unknown Ethnicity		263,867 1,840	18.427% 8.549%	<0.0001		1,827 1,840	8.488% 8.549%	0.8224
2131-1	Unknown Race		147,319 1,774	10.288% 8.242%	<0.0001		1,785 1,774	8.293% 8.242%	0.8473
2135-2	Hispanic or Latino		131,925 1,445	9.213% 6.713%	<0.0001		1,426 1,445	6.625% 6.713%	0.7136
2028-9	Asian		27,600 395	1.927% 1.835%	<0.0001		379 395	1.761% 1.835%	0.5617
1002-5	American Indian or Alaska Native		4,410 88	0.308% 0.409%	<0.0001		77 88	0.358% 0.409%	0.3909
2076-8	Native Hawaiian or Other Pacific Islander		2,704 94	0.189% 0.437%	<0.0001		62 94	0.288% 0.437%	0.0103
**Codes**	**Diagnoses**	**Mean ± SD**	**Patients**	**% of Cohorts**	**p-Value**	**Mean ± SD**	**Patients**	**% of Cohorts**	**p-Value**
Z00-Z99	Factors Influencing Health Status & Contact with Health Services		944,720 21,395	65.972% 99.401%	<0.0001		21,396 21,395	99.405% 99.401%	0.9501
M00-M99	Musculoskeletal Disease		534,817 22,395	37.348% 70.763%	<0.0001		15,259 15,231	70.893% 70.763%	0.7666
I10-I16	Hypertension		364,738 12,786	25.471% 59.403%	<0.0001		12,814 12,786	59.534% 59.403%	0.7834
E08-E13	Diabetes Mellitus		159,565 6,291	11.143% 29.229%	<0.0001		6,250 6,291	29.037% 29.228%	0.6636
E65-E68	Overweight/Obesity		151,851 5,155	10.604% 23.95%	<0.0001		5,152 5,155	23.936% 23.95%	0.9730
I25	Heart Disease		96,135 4,231	6.713% 19.957%	<0.0001		4,243 4,231	19.713% 19.657%	0.8843
I50	Heart Failure		62,874 3,162	4.398% 14.691%	<0.0001		3,082 3,162	14.319% 14.691%	0.2735
J44	COPD		55,531 2,906	3.878% 13.501%	<0.0001		2,891 2,906	13.432% 13.501%	0.8323

Cohorts were balanced across numerous demographic and diagnostic criteria using ICD-10 codes. Note that all variables were significantly different prior to propensity score matching, whereas all variables (except for Native Hawaiian/Pacific Islander racial group) did not significantly differ following propensity score matching.

**Table 3:** Risks of post-operative adverse outcomes within 30, 60, 90, and 120 days of surgery in SARS-CoV-2-positive patients with or without influenza vaccination.

30 Days (N=21,790)					
**Outcome**	**No Vaccine**	**Vaccine**	**Risk Ratio**	**p-value**	**95% CI**
Sepsis	0.84%	0.59%	1.437	0.0027**	(1.133,1.823)
DVT	0.29%	0.19%	1.535	0.0351**	(1.027,2.292)
PE	0.28%	0.26%	1.093	0.6385	(0.755,1.583)
Death	1.54%	1.37%	1.125	0.136	(0.964,1.314)
ARDS	0.19%	0.15%	1.239	0.358	(0.784,1.959)
Stroke	0.27%	0.20%	1.331	0.1688	(0.885,2.002)
Acute MI	0.42%	0.28%	1.476	0.0223**	(1.055,2.064)
Dehiscence	0.20%	0.10%	1.988	0.0087**	(1.178,3.355).
SSI	0.17%	0.13%	1.312	0.2758	(0.803,2.146)
Seroma	0.05%	0.08%	0.61	0.1921	(0.288, 1.291)
Hematoma	0.19%	0.21%	0.898	0.6254	(0.584, 1.382)
**60 Days (N=21, 790)**					
**Outcome**	**No flu shot**	**Yes flu shot**	**Risk Ratio**	**p-value**	**95% CI**
Sepsis	1.13%	0.80%	1.406	0.001*	(1.146,1.723)
DVT	0.43%	0.26%	1.66	0.0033**	(1.179,2.338)
PE	0.38%	0.35%	1.092	0.5855	(0.793,1.504)
Death	2.11%	1.86%	1.132	0.0665	(0.992, 1.292)
ARDS	0.21%	0.18%	1.176	0.4547	(0.768, 1.801)
Stroke	0.37%	0.27%	1.369	0.0767	(0.966, 1.941)
Acute MI	0.59%	0.34%	1.727	0.0002*	(1.284,2.321)
Dehiscence	0.29%	0.15%	1.926	0.0022**	(1.257,2.949)
SSI	0.31%	0.19%	1.664	0.0099**	(1.125,2.46)
Seroma	0.08%	0.10%	0.808	0.5133	(0.427,1.532)
Hematoma	0.25%	0.28%	0.881	0.5084	(0.605, 1.282)
**90 Days (N=21,790)**					
**Outcome**	**No flu shot**	**Yes flu shot**	**Risk Ratio**	**p-value**	**95% CI**
Sepsis	1.32%	0.87%	1.517	<0.0001*	(1.251,1.839)
DVT	0.49%	0.31%	1.614	0.0026**	(1.178,2.212)
PE	0.44%	0.40%	1.088	0.5759	(0.809, 1.463)
Death	2.46%	2.07%	1.188	0.0065**	(1.049,1.345)
ARDS	0.24%	0.20%	1.206	0.3621	(0.806, 1.806)
Stroke	0.46%	0.32%	1.432	0.0258**	(1.043,1.968)
Acute MI	0.70%	0.38%	1.82	<0.0001*	(1.379,2.402)
Dehiscence	0.34%	0.17%	2.073	0.0003*	(1.386,3.1)
SSI	0.37%	0.21%	1.766	0.002**	(1.226,2.543)
Seroma	0.09%	0.11%	0.781	0.4319	(0.527,1.452)
Hematoma	0.29%	0.30%	0.966	0.8487	(0.676,1.381)
**120 Days (N=21,790)**					
**Outcome**	**No flu shot**	**Yes flu shot**	**Risk Ratio**	**p-value**	**95% CI**
Sepsis	1.44%	0.99%	1.461	<0.0001*	(1.218,1.753
DVT	0.55%	0.34%	1.607	0.0017**	(1.192,2.166)
PE	0.47%	0.42%	1.119	0.4466	(0.838, 1.493)
Death	2.71%	2.16%	1.257	0.0002*	(1.115,1.417)
ARDS	0.26%	0.20%	1.276	0.23	(0.856, 1.9)
Stroke	0.53%	0.33%	1.613	0.002**	(1.188,2.19)
Acute MI	0.80%	0.40%	2.015	<0.0001*	(1.542,2.633)
Dehiscence	0.39%	0.18%	2.115	<0.0001*	(1.427,2.886)
SSI	0.44%	0.22%	2.03	<0.0001*	(1.427, 2.886)
Seroma	0.10%	0.12%	0.879	0.6578	(0.495, 1.559)
Hematoma	0.31%	0.33%	0.937	0.7081	(0.666, 1.317)

95% CI = 95% Confidence Interval

* denotes statistical significance following Post-Hoc testing for Multiple Hypotheses using Bonferroni Correction to alpha level of p = 0.0011

** denotes nominally significant values (p < 0.05) that failed to meet statistical significance following Bonferroni correction of p = 0.0011.

Subsequently, Absolute Risk Reduction (ARR), defined as the difference in risk of adverse post-operative outcomes between the influenza vaccinated group versus non-vaccinated group was calculated for each adverse outcome. The reciprocal of ARR was then obtained to determine number needed to treat, defined in this study as number needed to vaccinate (NNV), for all statistically significant variables at 30–120 days post-operatively. The NNV calculation allowed for the quantification of the average number of SARS-CoV-2-positve patients who needed to be vaccinated against influenza two weeks to six months prior to undergoing surgery in order to prevent one adverse post-operative outcome [18, 19].

## Results

A total of 1,435,293 SARS-CoV-2-positive surgery patients met inclusion criteria and were assigned to one of two cohorts based upon influenza vaccination status. 1,413,312 patients were not up-to-date on their influenza vaccination prior to undergoing any type of surgery whereas 21,981 were current (Table 1). Between-group factors for all propensity scoring categories were found to be significantly different (p<0.0001) prior to cohort balancing. Following propensity score matching, the differences between cohorts were no longer significant (p>0.05), with the exception of a significant difference in the number of Native Hawaiian/Other Pacific Islander individuals (p = 0.0103) (Table 2). After matching, 43,580 patients remained and were divided amongst two equally-sized cohorts of 21,790 patients based upon influenza vaccination status. Each group contained equivalent proportions of females to males, with 59.5% of patients being female and 40.5% being male. The mean age of the influenza vaccinated group was 58.9 years while the non-influenza vaccinated group had a mean age of 59.0 years.

**Fig 3:**
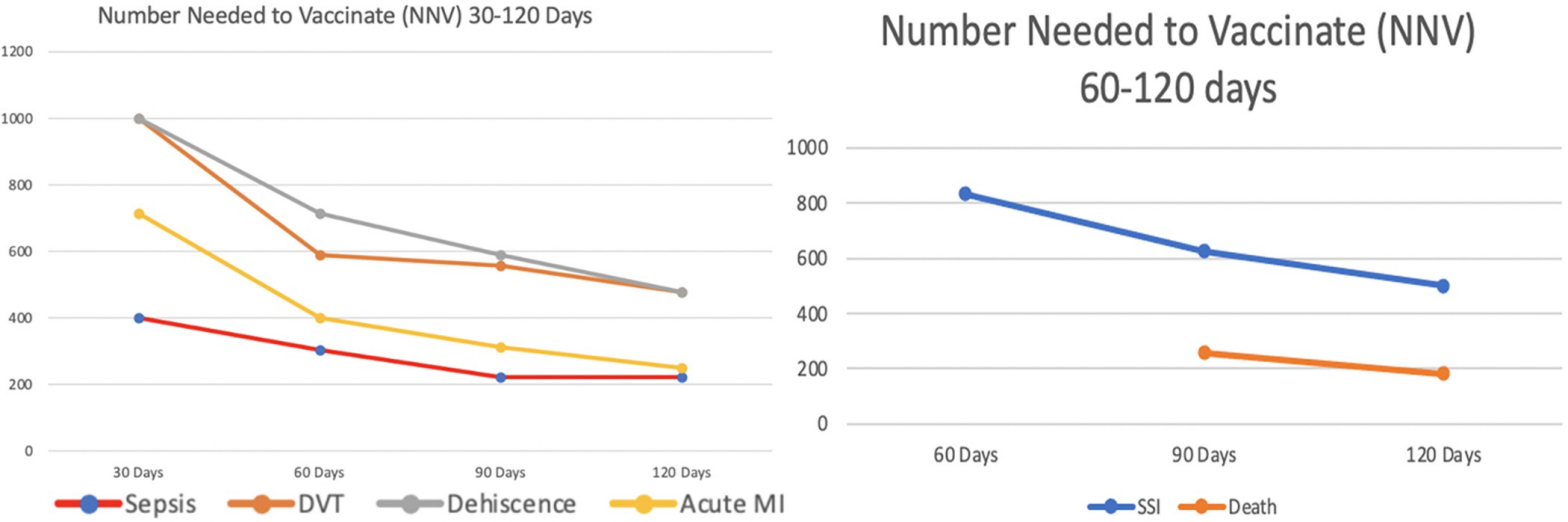
Number needed to vaccinate with influenza immunization to prevent one of the following post-operative adverse outcomes within 30–120 days (Fig 3a) and 60–120 days (Fig 3b) of surgery in this population.

When compared to SARS-CoV-2-positive patients without influenza vaccination prior to surgery, SARS-CoV-2-positive patients with influenza vaccination prior to surgery experienced significantly decreased risk of sepsis within 60–120 days post-operatively with nominal significance within 30 days [(p = 0.0001–0.0027, RR = 1.437–1.517, 95% CI:1.133–1.823) NNV:223–400], significantly decreased risk of acute MI within 60–120 days post-operatively with nominal significance within 30 days [(p = 0.0001–0.0223, RR = 1.476–2.015, 95% CI:1.055–2.064) NNV: 250–715], significantly decreased risk of dehiscence within 90–120 days post-operatively with nominal significance within 30 and 60 days [(p = 0.0001–0.0087, RR = 1.988–2.03, 95%CI:1.178–3.355) NNV: 715–1000], and nominally significant decreased risk of DVT within 30–120 days post-operatively [(p = 0.0017–0.0351, RR = 1.535–1.607, 95% CI:1.027–2.292) NNV: 476–1000], and (Table 3, Figs 2 & 3).

Additionally, SARS-CoV-2-positive patients up-to-date on their influenza vaccination prior to surgery experienced a significant reduction in death within 120 days post-operatively with nominal significance at 90 days [(p = 0.0002–0.0065, RR = 1.188–1.257, 95% CI:1.049–1.345) NNV: 256–682] and had significantly fewer SSIs within 120 days post-operatively with nominal significance within 60 and 90 days [(p = 0.0001–0.0099, RR = 1.66–2.03, 95% CI:1.125–2.46) NNV: 500–833] (Table 3, Figs 2 & 3).

## Discussion

To the best of our knowledge, this study is the first to examine the potential protective effects of influenza vaccination against post-operative complications in SARS-CoV-2-positive patients undergoing any surgical procedure. Our analysis also underscores the possible utility of a global, federated EMR network during worldwide health crises.

The de-identified EMRs included in our study were evaluated for adverse outcomes at 30, 60, 90, and 120 days post-surgery (Fig 1). The upper limit of our study’s timespan was set at 120 days in order to account for the possible presence of Post-Acute COVID-19 Syndrome (PACS). PACS is associated with increased risk of illness-related fatigue, dyspnea, inflammation, and neurologic symptoms. Given previous reports of substantial improvement 16–18 weeks after onset, our 120-day endpoint ensured capture of this phenomenon [20–24].

The potential protective effect of influenza vaccination against adverse outcomes associated with SARS-CoV-2 infection has been well-documented in non-surgical patients [25–31]. One of the first studies to demonstrate this correlation was a single-center retrospective by Yang et al. [6]. This study found a significant reduction in hospitalization and ICU admission in influenza-vaccinated patients. Shortly after, Conlon et al. supported this finding, demonstrating that SARS-CoV-2-positive patients immunized against influenza experienced decreased risks of hospitalization, mechanical ventilation, and length of stay [7]. Another key finding was put forth in a retrospective analysis that observed a significantly decreased risk of death in COVID-positive patients current on their influenza immunization [32]. Given our present finding of significant and nominally significant reductions in risk of sepsis, DVT, Acute MI, and dehiscence across all time points 30–120 days (Fig 2A), it appears that the potential protection afforded by influenza vaccination against SARS-CoV-2 may extend to surgical patients in the postoperative period. Further supporting this proposed protective effect are the additional significantly decreased risks of SSIs and death within 120 days following surgery (Fig 2B, Table 3)

SARS-CoV-2-positive surgical patients that underwent surgery are more vulnerable to adverse outcomes given the inflammatory and catabolic nature of surgery [33, 34]. Specifically, post-operative patients release an exorbitant amount of pro-inflammatory cytokines along with increased levels of cortisol [35]. This systemic inflammation, when considered alongside the baseline hypercoagulable state induced by endothelial cell invasion by COVID-19, presumably compounds risk of poor post-operative outcomes in the surgical patient population [36].

Although the exact manner by which influenza vaccination confers protection against adverse post-operative outcomes in SARS-CoV-2-positive surgical patients remains undetermined, several mechanistic theories have been hypothesized [9–15]. The proposed mechanisms converge on the premise that the influenza vaccine appears to stimulate the body’s innate immune system, thereby interfering with SARS-CoV-2 replication. One of these theories suggests that influenza immunization activates Toll-Like-Receptor-7 on cells, thereby impeding replication of single-stranded RNA viruses [11]. Alternatively, it has been proposed that influenza vaccination may prime natural killer cells, thus increasing innate immunity to combat viral antigens [14].

Another investigation demonstrated that immunization against influenza upregulates pulmonary angiotensin-converting enzyme 2 (ACE-2) receptors [10]. Downregulation of ACE-2 receptors, (observed in SARS-CoV-2-positive patients) induces pulmonary inflammation and coagulation as fewer angiotensin-II can bind to antioxidant ACE-2 receptors. As a result, the angiotensin-II instead binds to pro-inflammatory angiotensin I receptors resulting in systemic inflammation [15].

Two final postulates are notable in the context of the present study. Firstly, influenza vaccination stimulates an immunologic cascade leading to plaque stabilization, thus, decreasing risk of cardiovascular events. Vaccine-induced antibodies interact with bradykinin-2 receptors leading to increased nitric oxide production and an anti-inflammatory effect [9]. Lastly, the preparation of the trivalent influenza vaccine uses an oil-in-water squalene emulsion, known as MF59, that may induce protection against SARS-CoV-2 via host immune system stimulation [12].

Regardless of mechanism, the strong association between influenza vaccination and decreased risk of adverse post-operative outcomes in SARS-CoV-2-positive patients observed by this study merits further investigation. The hypothesis that individuals current on their influenza immunization have less baseline medical co-morbidities and risk factors for poor post-operative outcomes is supported by the “Before Matching” column in Table 2. In addition, recent literature has suggested that certain characteristics including age, gender, and BMI may dictate SARS-CoV-2 outcomes [37–43]. However, Table 2 also demonstrates the results of the stringent propensity score matching performed, accounting for numerous characteristics, which would have otherwise potentially acted as confounders.

By contextualizing study findings on a global scale, NNV has been an extremely valuable tool for effect size analysis throughout the SARS-CoV-2 pandemic to measure the protective effects of both influenza and coronavirus vaccines [8, 44]. Specifically, our NNV calculations revealed that within 120 days, 223, 250, 323, and 182 individuals would have needed to have been current on their pre-operative influenza immunization to avoid one case of sepsis, acute MI, pneumonia, and death, respectively. Given, the potential benefits elucidated by NNV calculations, ramping up influenza vaccination in parallel with COVID-19 vaccination merits strong consideration.

Even with the unprecedented fast-tracking of multiple vaccines, the fact remains that a majority of the world is not fully vaccinated against SARS-CoV-2 [45]. Furthermore, current projections suggest that numerous countries may not receive sufficient Covid-19 vaccines for years, as partitioning continues to favor nations with the highest gross domestic products [46, 47]. Given the delay of equitable access to the Covid-19 vaccine for the global community, there remains a need for preventative measures to attempt to curb disease burden in affected patients. Influenza vaccination maintains a status as a well-accepted and abundantly available option for the global community. Its low cost and predictable side effect profile make influenza vaccination a valuable provisional option to consider for individuals lacking access to COVID-19 vaccines, and may yield a benefit for individuals with predicted high risk of surgical mortality [43, 48]. Influenza vaccination may also prove useful for citizens with coronavirus vaccine access, but a hesitancy to consent to novel vaccines [49, 50]. Thus, pre-operative influenza vaccination may be beneficial in reducing SARS-CoV-2 morbidity and mortality in post-operative patients world-wide [51–53]. Furthermore, the global population may benefit from influenza vaccination as it can act to prevent a coronavirus and influenza ‘twindemic’ which could overwhelm healthcare resources [54].

This study is limited by its retrospective nature and reliance on the accuracy of medical coding. This study is also limited by its time window of January 2020-January 2021, prior to the widespread availability of SARS-CoV-2 vaccination, thereby prohibiting analysis of any potential synergistic or interactive effects between the influenza and COVID-19 vaccines in this patient population. Federated EMR networks lend themselves to measures of association, but not causation, thus future prospective studies are warranted to validate this study’s finding that an emphasis on influenza vaccination will improve post-operative outcomes in COVID-positive surgery patients.

## Conclusion

Using a federated EMR network of over 73 million patients globally, this analysis examines the potential protective effect of influenza vaccination against adverse post-operative outcomes within 30, 60, 90, and 120 days of SARS-CoV-2-positive surgery patients. Significant findings in favor of the influenza vaccine in mitigating the risks of sepsis, acute MI, and dehiscence across all multiple time points while decreasing the risk of SSI and death by 120 days suggest a potential protective effect that merits further investigation and validation with prospective studies, such as randomized control trials.
